# Brain Natriuretic Peptide (BNP) Affects Growth and Stress Tolerance of Representatives of the Human Microbiome, *Micrococcus luteus* C01 and *Alcaligenes faecalis* DOS7

**DOI:** 10.3390/biology11070984

**Published:** 2022-06-29

**Authors:** Nataliya Loiko, Oleg Kanunnikov, Andrei Gannesen, Vladislav Kovalenko, Anastasia Vishnyakova, Vladimir Axelrod, Yuriy Litti

**Affiliations:** 1 Winogradsky Institute of Microbiology, “Fundamentals of Biotechnology” Federal Research Center, Russian Academy of Sciences, 117312 Moscow, Russia; andrei.gannesen@gmail.com (A.G.); nast366760404@mail.ru (A.V.); 2 Rail Chemical LLC, 105005 Moscow, Russia; ole1256@gmail.com (O.K.); v_axe@mail.ru (V.A.); 3 Semenov Federal Research Center for Chemical Physics, Russian Academy of Sciences, 119991 Moscow, Russia; vladislavkovalenko785@gmail.com

**Keywords:** brain natriuretic peptide (BNP), *Micrococcus luteus*, *Alcaligenes faecalis*, stress resistance, osmotic stress, acid and base stress, oxidative stress, thermal stress, antibiotic stress, long-term survival

## Abstract

**Simple Summary:**

The body of an average person weighing 70 kg contains approximately 39 trillion bacterial cells, which densely inhabit the gastrointestinal tract, skin, mucous membranes, etc. Bacteria respond to the signaling molecules in the human body, regulate the expression of the necessary genes, and thus adapt to the physiology of the host. Signaling molecules include hormones, neurotransmitters, immune system molecules, as well as natriuretic peptides, which are involved in the regulation of the circulatory system, water and electrolyte metabolism, and adipose tissue metabolism. Brain natriuretic peptide (BNP) is secreted by the ventricles during congestion and signals heart failure. This study showed that the presence of BNP in the growth medium of human symbiont bacteria affects their growth characteristics, survival, and stress resistance, including antibiotic resistance. It was concluded that bacterial populations that develop in a healthy person at a BNP level of up to 250 pg/mL will be more stress resistant than in a person suffering from heart failure. Our findings are promising to be used both in clinical medical practice and in the production of bacterial preparations for cosmetology, agriculture, and waste management.

**Abstract:**

Brain natriuretic peptide (BNP) is secreted by the ventricles of the heart during overload to signal heart failure. Slight bilateral skin itching induced by BNP has been associated with response activity of the skin microbiota. In this work, we studied the effect of 25–250,000 pg BNP/mL on the growth, long-term survival, and stress (H_2_O_2_, antibiotics, salinity, heat and pH shock) resistance of human symbiont bacteria: Gram-positive *Micrococcus luteus* C01 and Gram-negative *Alcaligenes faecalis* DOS7. The effect of BNP turned out to be dose-dependent. Up to 250 pg BNP/mL made bacteria more stress resistant. At 2500 pg BNP/mL (heart failure) the thermosensitivity of the bacteria increased. Almost all considered BNP concentrations increased the resistance of bacteria to the action of tetracycline and ciprofloxacin. Both bacteria survived 1.3–1.7 times better during long-term (up to 4 months) storage. Our findings are important both for clinical medical practice and for practical application in other areas. For example, BNP can be used to obtain stress-resistant bacteria, which is important in the collection of microorganisms, as well as for the production of bacterial preparations and probiotics for cosmetology, agriculture, and waste management.

## 1. Introduction

The term microbial endocrinology was suggested for describing the phenomena of eukaryotic hormone recognition by microbes in Mark Lyte’s innovative work in 1993 [1]. There are numerous observations where the connection between human stress hormones and pathogenic infections has been outlined [2]. It was shown that eukaryotic signal molecules (or so-called humoral regulation factors, HRF) such as hormones, neurotransmitters, immune system molecules, etc. could modulate physiological behavior and Gram-negative pathogens’ growth [3,4], and also influence its virulence [5] and biofilms formation [6,7]. Microorganisms found a way to adapt to host physiology by responding to its HRF through the regulation of expression time of necessary genes [2].

Studies of the last decade show that all human microbes are involved in the process of signal communications. Microorganisms can not only respond to eukaryotic signal molecules, but also express a variety of host HRF, for example, during stress. As a result, a new field of microbial endocrinology has emerged, which studies the bidirectional mechanisms of signaling interaction between the microbiota and humans [8,9]. Undeniable evidence shows that a microorganism’s signal molecules play a significant role in the regulation of the endocrine, immune, vegetative, intestinal, and nervous systems of humans and animals. The similarity of most HRF biosynthesis pathways in humans and microbiota was established, which indicates its acquisition by microorganisms during lateral gene transfer [10]. A typical example is the complex bi-directional connection between the intestine and the brain, regulated by the peptide interactions of the intestinal microflora. Disruption of these interactions leads to various brain dysfunctions such as anxiety and depression [11,12].

It is important to study the regulatory interactions of microorganisms that form complex multispecies biofilms on human skin [6,13,14,15,16]. The skin is the human body’s largest organ, containing hundreds of microbial genera [17]. It plays a significant role in the adaptation of the body’s physiology to environmental changes, including its ability to express a wide range of signal molecules (hormones, neurotransmitters, and cytokines) [13]. At the same time, local microenvironmental changes often proceed on human skin, which leads to variation in HRF contained in a wide range of concentrations [6,14].

A substantial contribution to human-microbial interaction has been made by natriuretic peptides (NP), which are involved in blood supply system regulation, hydro-electrolytic metabolism, fat tissue metabolism, etc. [18]. In our latest works, it has been shown that two natriuretic peptides—atrial natriuretic peptide (ANP) and C-type natriuretic peptide (CNP)—can have a considerable impact on skin microbiota growth, and also regulate the activity and formation of monospecific and mixed biofilms [6,14]. The third important NP is a brain natriuretic peptide (BNP). It is secreted by heart ventricles during its overload for signaling heart failure [19]. There is a lack of information about its effect on skin microbiota.

BNP was first purified from a pig’s brain in 1988 [20]. Subsequent experiments have shown that BNP is produced in mammals not only by the brain but also by cardiac myocytes and has the same receptor apparatus as an ANP [21]. All of the NPs are synthesized as prohormones and then decompose into bioactive C-terminus forms and N-terminus residues. BNP precursors (pro-BNP, 108 amino-acid residues) decompose to active BNP (32 amino-acid residues, half-life period is 20 min), and biological inertial N-terminus fragment pro-BNP (NT-pro-BNP, 76 amino-acid residues, half-life period is 120 min) under the influence of specific ferment—furin [22,23].

The systematic effect of the BNP is well described [23,24]. It is secreted as a response to the mechanical expansion of the myocardial walls, caused by overload or pressure. It also exerts diuretic, natriuretic, and vasodilatory effects, thereby maintaining cardiorenal homeostasis and hemodynamic status by regulating electrolyte balance and fluid volume in the body [25]. The effects of BNP during both steady-state and pathological conditions are realized through a complex of physiological reactions at the level of various body systems, such as cardiovascular, excretory, endocrine, and central nervous system (CNS). Blood BNP concentration has variability by age and gender: it increases with age and women have a higher value of it [26]. The upper limit of the physiological norm for BNP is considered to be 100 pg/mL, regardless of age, and for NT-proBNP it is 125 pg/mL for people under 75 years old, and 450 pg/mL—for people older than this age [22]. A total of 90% of young healthy patients have a blood BNP concentration not exceeding 25 pg/mL, and NT-proBNP at 70 pg/mL [27,28].

The concentration of BNP and NT-proBNP in the blood is an important diagnostic test for heart dysfunction in a wide variety of clinical situations, as they significantly increase in chronic myocardium, atrium, and ventricles distention, for example, during chronic blood circulation insufficiency. In studies conducted by the Dorofeykov group, it has been shown that patients’ plasma BNP levels with chronic heart failure of the fourth functional class can reach 2600 pg/mL [29]. The clinical determination of BNP in the patient’s blood allows for the determination of the severity and predicting the further course of many pathological conditions, as well as to evaluate the effectiveness of the treatment of cardiovascular diseases [30,31]. BNP can also signal other changes in the body, such as cognitive dysfunction or dementia [32], and affect various organs, including human skin [33]. Mishra S.K. and Hoon M. A. showed in their studies on mice that BNP acts as a specific biomarker transmitting signals between the CNS and the skin and causing itchiness [34]. Other researchers, even though refuting the mechanisms of action proposed by Mishra’s group, have confirmed its direct involvement in the induction of skin itching. They showed that injecting mice with 1 nmol (4000 pg/mL) BNP caused slight bilateral skin itching during the first 30 min and increased the force of this effect in the next 30 min [35]. This experiment proves that microorganisms that inhabit the skin surface have experienced strong stress which undoubtedly affects their metabolism and behavior when levels of BNP in the blood increase [33].

The clinical symptoms and mechanism of action of BNP are well documented in medical research. However, there is a lack of studies devoted to how BNP affects microorganisms. Deciphering all aspects of human–microbiota signal interactions via HRF is essentially important for creating new strategies for the prevention and treatment of infectious diseases [36] and also allows us to design next-generation drugs.

The aim of this work was to study the effect of the natriuretic peptide BNP on the growth and stress resistance of the human microbiota. Based on the promising results of previous studies on the effect of hormones on vital functions and biofilm formation [15,37,38,39] and some beneficial biotechnological features (for use in cosmetology, agriculture, and waste management), two bacteria, *Micrococcus luteus* C01 and *Alcaligenes faecalis* DOS7, were selected as Gram-positive and Gram-negative representatives of the human microbiome.

## 2. Materials and Methods

### 2.1. Bacteria and Natriuretic Peptide

The objects of the study were Gram-positive bacteria *Micrococcus luteus* C01, isolated from human skin [15] and Gram-negative bacteria *Alcaligenes faecalis* DOS7, isolated from fecal waste.

The Natriuretic Peptide (BNP) (Merck, Darmstadt, Germany) was diluted from stock aqueous solution with double distilled water to the desired concentrations just prior to the experiment. The stock solution was stored at a temperature of −18 °C.

### 2.2. Cultivation

Bacteria were grown in Luria-Bertani (LB) (Broth, Miller, VWR, Radnor, Pennsylvania, USA) medium. Inoculum was the early stationary phase culture with an initial concentration of 8.0 × 10^7^ cells/mL. Cultivation was carried out in 2 mL microtubes with 1 mL of medium by stirring on a thermoshaker (BIOSAN TS_100S, Riga, Latvia) at a speed of 750 rpm at 34 °C (*M. luteus*) or 37 °C (*A. faecalis*).

Before cultivation, BNP was added once to the experimental populations to the desired concentration: 25,250, 2500, 25,000, and 250,000 pg/mL.

Cell viability was determined by the number of colony-forming units (CFU) when cell suspensions were seeded from appropriate dilutions on LB agar medium (with the addition of 3% Bacteriological Agar, Helicon, Moscow, Russia).

The growth of bacterial populations was controlled by the change in the optical density of the suspension, which was measured at 595 nm on a Spectrophotometer 7315 (Jenway, Stone, Staffordshire, UK).

### 2.3. Storage

Bacterial populations were stored in their growth medium in plastic test tubes without air access at a temperature of 21 °C for 4 months. The number of viable cells was determined periodically.

### 2.4. Effect of BNP on Bacterial Activity

BNP effects on bacterial activity were studied under aerobic and microaerophilic conditions in tightly closed 120 mL glass vials with 10 and 25 mL of medium, respectively. Before incubation, BNP was added to the experimental populations at concentrations of 25, 250, 2500, 25,000, and 250,000 pg/mL. In the experiment under aerobic conditions, an additional 40 mL of air was added to each vial to avoid oxygen limitation. Bacterial activity was assessed by accumulation of CO_2_ in the gas phase, and the presence of acetate in the medium was additionally identified for microaerophilic conditions.

### 2.5. Assessment of Stress Tolerance

The thermostability of bacterial cells’ exponential and stationary growth phase was determined by their survival after warm-up of cell suspension (450 µL) for 15 min in a thermoshaker (BIOSAN TS_100, 450 rpm, Latvia) at a temperature of 60 °C, followed by determining the number of viable cells by counting CFU on an LB agar plate.

The resistance of bacterial cells’ exponential growth phase to osmotic stress was determined by their survival after incubation in 33% NaCl aqueous solution for 2 h, followed by determining the number of viable cells by counting CFU on an LB agar plate.

The resistance of bacterial cells’ exponential growth phase to acidic and alkaline (base) stress has been determined by their ability to maintain vitality after incubation on media at pH 3.5 or pH 10.5 (1N HCl or NaOH were added) for 24 h, followed by determining the number of viable cells by counting CFU on an LB agar plate.

The resistance of bacterial cells’ exponential growth phase to oxidative stress was determined by their survival after incubation in the presence of 0.3% H_2_O_2_ within 12 min, followed by determining the number of viable cells by counting CFU on an LB agar plate.

### 2.6. Determination of Minimum Inhibitory Concentrations (MIC)

Two mL of LB medium and aqueous solutions of ciprofloxacin and tetracycline antibiotics with the final concentrations of 25, 250, 2500, 25,000, and 250,000 pg/mL were added to the 15 mL glass tubes. The tubes were then inoculated by a two-day stationary growth phase culture, pre-cultivated with BNP (experimental) or without it (control). The tubes were incubated in a thermostatted shaker at 28 °C. After 4 days, the growth of bacteria was visually assessed in the presence of different antibiotic concentrations. The smallest antibiotic concentration, at which no bacterial growth was observed, was established as MIC.

### 2.7. Analytical Methods

The CO_2_ concentration was measured on a gas chromatograph (GC) (Crystal 5000.2, Chromatek, Yoshkar-Ola, Russia) as described earlier [40]. Acetic acid was determined by a GC (Crystal 5000.2, Chromatek, Russia) as described earlier [40]. Briefly, the DB-FFAP column (30 m _ 0.32 mm _ 0.25 μm) was used for separation. The oven temperature was increased from 100 to 165 °C at a rate of 8 °C/min; injector and flame ionization detector temperatures were set at 220 and 230 °C; carrier gas (argon) flow rate was 20 mL min^−1^. The pH values of the samples were adjusted to 2 with formic acid. Acidified samples were centrifuged at 10,000 rpm for 5 min and analyzed by GC. Acetic acid was calibrated using the internal standard which was 2-ethyl butyric acid (99% purity, Sigma-Aldrich, Saint Louis, MI, USA).

### 2.8. Statistical Methods

All experiments were performed in triplicate. Statistical analysis was carried out using standard mathematical methods (Student’s *t*-test and calculation of the standard deviation) using the Microsoft Excel program. The data group was considered homogeneous if the mean square deviation σ did not exceed 10 per cent. The differences between the data groups were considered valid under the probability criterion *p* < 0.05.

## 3. Results

**Figure 1:**
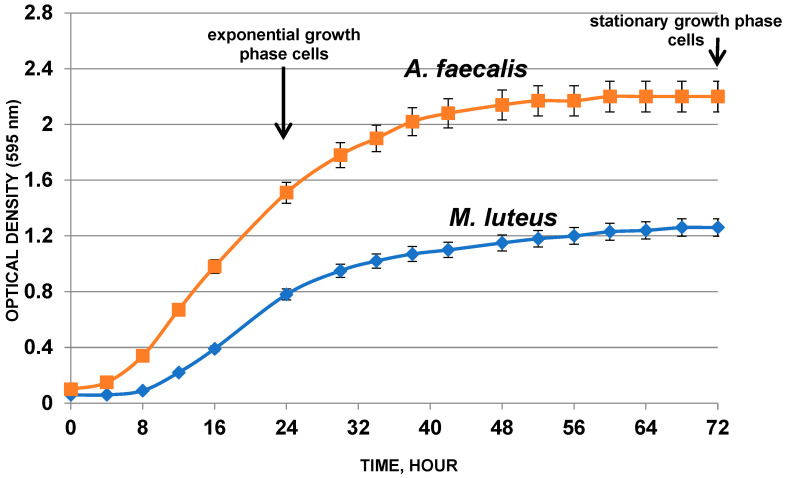
Growth curves of control populations of *A. faecalis* and *M. luteus*.

Gram-positive bacteria *Micrococcus luteus* C01 isolated from human skin [15,37,38,39] and Gram-negative bacteria *Alcaligenes faecalis* DOS7 isolated from fecal waste were used as representatives of the human microbiota. These bacteria were cultivated under microaerophilic conditions in 2 mL closed plastic tubes with 1 mL of medium with stirring at a temperature of 34 °C (*M. luteus*) or 37 °C (*A. faecalis*). A graphical representation of the growth curves of the control populations *M. luteus* and *A. faecalis* is shown in Figure 1. Experimental populations were cultured in the presence of BNP with concentrations of 25, 250, 2500, 25,000, and 250,000 pg/mL. This experiment imitated bacterial (skin or intestinal origin) growth in a healthy human body or a body suffering from chronic heart failure. The smallest BNP concentration was 25 pg/mL (referred to as N (normal, physiological)). This value corresponds to the average physiological concentration of this hormone in the blood of a healthy person. Higher concentrations of the peptide, in particular 250 pg/mL (referred to as 10 N) and 2500 pg/mL (referred to as 100 N), correspond to the concentration of the peptide in the blood of patients with chronic heart failure [29,41,42]. Studies with bacterial population grown in the presence of higher concentrations of peptide (25,000 pg/mL (referred to as 1000 N) and 250,000 pg/mL (referred to as 10,000 N)) were carried out to understand the mechanisms of regulation of the action of this hormone on microorganisms, as well as to identify its potential in the development of new drugs.

### 3.1. The Effect of BNP on the Growth and Survival of Bacteria during Long-Term Storage

**Figure 2:**
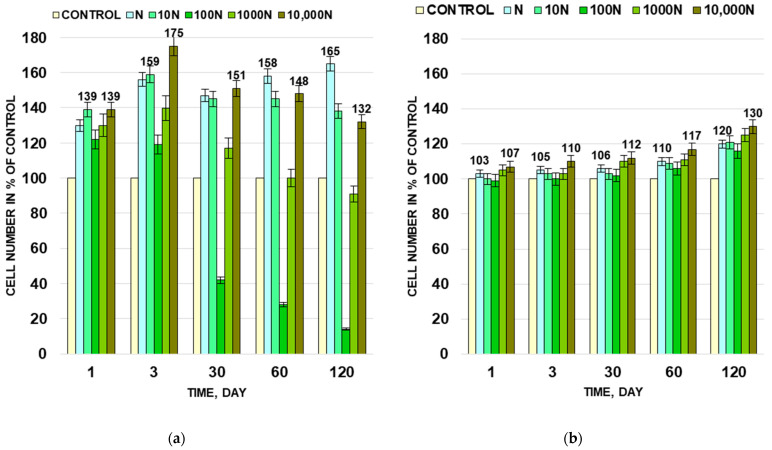
The effect of BNP on the cell number of *M. luteus* (**a**) and *A. faecalis* (**b**) in experimental populations in exponential (1 day) and stationary (3 days) growth phases and in long-term storage (30, 60, and 120 days).

It was discovered that the presence of BNP affects the growth of Gram-positive *M. luteus* and has almost no effect on the growth of Gram-negative bacteria *A. faecalis*. (Figure 2). All five experimental populations of *M. luteus* with different concentrations of BNP have a decrease in lag phase and increase of the cell number during the log (24 h) and stationary (72 h) phases of growth compared to the control population (Figure 2a). The strongest effect of BNP was observed in the 10 N and 10,000 N populations. The number of CFU in these populations after 24 h of incubation was 1.4 times higher than in the control, and after 72 h it increased by 1.6 and 1.7 times for the 10 N and 10,000 N populations, respectively. For experimental populations of *A. faecalis* there were no changes in lag phase duration, and a slight increase of the cell number in the stationary growth phase was observed in the N, 10 N and 10,000 N populations (Figure 2b).

The effect of BNP on the number of surviving cells was discovered during long-term storage of *M. luteus* and *A. faecalis* populations. The cell number in the control population of *M. luteus* decreased by 18.5 times from (6.3 ± 0.3) × 10^8^ to (3.4 ± 0.2) × 10^7^ CFU/mL during 4 months of storage. In N, 10 N and 10,000 N populations, the cell number after 4 months was 165, 147, and 165% of the control population (Figure 2a). In contrast, for the 100 N population, the cell number decreased to (4.8 ± 0.2) × 10^6^ CFU/mL after 120 days of storage, which is only one seventh part of the number of CFU in the control population. For the 1000 N population, the cell number after 30, 60, and 120 days of storage did not differ significantly from the control population.

In the case of Gram-negative bacteria *A. faecalis*, a different trend was observed. During long-term storage, the cell number decreased approximately by one order of magnitude from (1.5 ± 0.1) × 10^9^ to (1.2 ± 0.1) × 10^8^ CFU/mL. No significant differences in cell numbers between all experimental and control populations of that strain were observed. After 4 months of storage, these differences were 20, 21, 16, 25, and 30% for N, 10 N, 100 N, 1000 N, and 10,000 N, respectively, compared with the control (Figure 2b).

Obtained patterns of BNP effects on bacterial growth were confirmed in the next experiment with *M. luteus* and *A. faecalis* bacteria grown under aerobic and microaerophilic conditions in tightly closed 120 mL glass vials with 10 and 25 mL of medium, respectively. In the experiment under aerobic conditions, an additional 40 mL of air was added to each vial to avoid oxygen limitation. Bacterial activity was assessed by accumulation of CO_2_ in the gas phase, and the presence of acetate in the medium was additionally identified for microaerophilic conditions (acetate did not accumulate in aerobic conditions) (Table 1).

For experimental populations of *M. luteus* under aerobic conditions, a significant increase in CO_2_ content was found, especially for 1000 N and 10,000 N. For *A. faecalis*, the differences in the CO_2_ content in the experimental and control populations did not exceed the statistical errors. Under microaerophilic conditions, differences in the content of CO_2_ and acetate were recorded for both Gram-positive and Gram-negative bacteria. In both cases, CO_2_ and acetate concentrations were the highest in the 10 N population. However, the increase of acetate content in the 10 N population of *M. luteus* was about 60% of the control, and in the 10 N population of *A. faecalis* acetate content was higher by 7.9 times compared to the control population.

Thus, our experiments show that the presence of BNP affects the growth of bacteria, the accumulation of metabolites, the number of viable cells, and their long-term storage survival. Influence of BNP is shown to be stronger for Gram-positive *M. luteus* bacterial cells. The strength and direction of an effect depends not only on BNP concentration, but also on the growth conditions.

### 3.2. Effect of BNP on the Thermostability of Bacteria

One of the important indicators of a cell’s stress-tolerance is their ability to endure high temperature exposure. In the present study, populations of *M. luteus* and *A. faecalis* in their exponential and stationary growth phases were exposed to 60 °C for 15 min to evaluate how BNP affected bacterial heat tolerance. After that exposure, the cell number in the control population of *M. luteus* at the exponential growth phase decreased by two orders of magnitude from (2.3 ± 0.1) × 10^8^ to (3.5 ± 0.2) × 10^6^ CFU/mL, and at stationary phase about one and a half orders of magnitude from (6.3 ± 0.2) × 10^8^ to (9.3 ± 0.3) × 10^6^ CFU/mL. Gram-negative bacteria *A. faecalis* were more thermal-sensitive. A 15 min exposure to thermal stress decreased the population in an exponential growth phase by almost five orders of magnitude from (7.8 ± 0.3) × 10^8^ to (1.1 ± 0.1) × 10^4^ CFU/mL, and in stationary phase by four and a half orders of magnitude from (1.5 ± 0.2) × 10^9^ to (4.1 ± 0.3) × 10^4^ CFU/mL.

**Figure 3:**
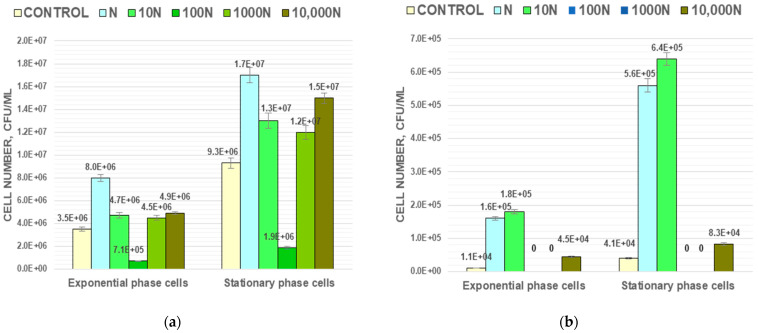
The effect of BNP on the thermal stability of *M. luteus* (**a**) and *A. faecalis* (**b**) cells in exponential and stationary growth phases (15 min exposure to 60 °C).

Cells grown under BNP treatment differed in thermosensitivity. The N, 10 N and 10,000 N populations for both strains were more heat tolerant than the control populations, while the 100 N population was less heat tolerant (Figure 3). For 1000 N populations, the result of observed thermosensitivity was the opposite for Gram-positive and Gram-negative bacteria. The pattern was similar for cells of different physiological ages. Differences in thermosensitivity between the control and experimental populations were more pronounced for Gram-negative bacteria. For example, the survival rate for the N and 10 N populations was one order of magnitude higher than for the control, but the 100 N and 1000 N populations did not give CFU at all at the end of thermal stress exposure (Figure 3b). The difference in thermosensitivity between control and experimental populations of *M. luteus* was less drastic (Figure 3a). The N population at the exponential phase of growth had the highest number of cells that survived heat stress. It was 2.3 times higher than in the control. The 10 N, 1000 N, and 10,000 N populations had 1.3–1.4 times higher cell numbers compared to the control, but in the 100 N population the cell number was five times lower compared to control. Similar observations were made for stationary phase populations.

### 3.3. The Effect of BNP on Bacterial Cell Resistance to Osmotic, Oxidative, Acid, and Base Stresses

**Figure 4:**
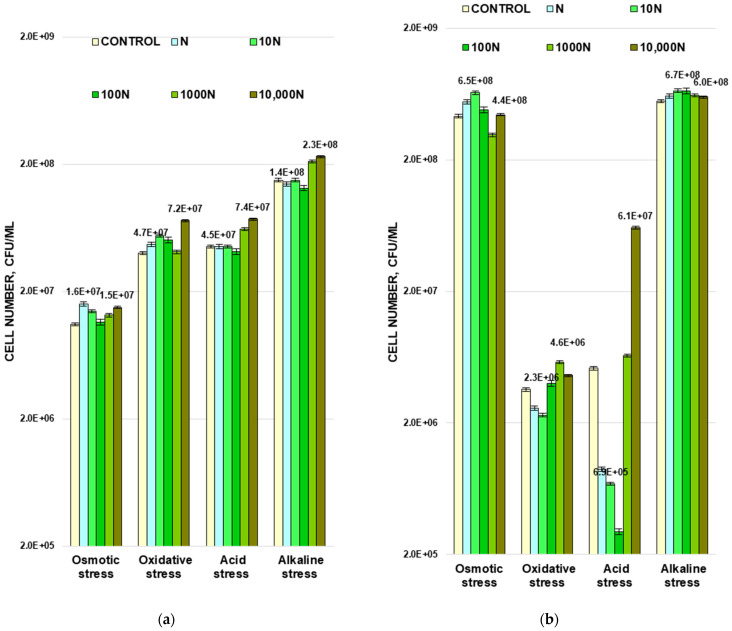
The effect of BNP on the resistance of *M. luteus* (**a**) and *A. faecalis* (**b**) cells to osmotic, oxidative, acidic, and alkaline stresses.

The next step was to study the differences in cell viability after exposure to the control and experimental populations of *M. luteus* and *A. faecalis* in the phases of their exponential growth of four types of stress: osmotic, oxidative, acid, and base (Figure 4).

Bacterial cells are exposed to each type of these stresses during growth in the human body. For example, skin during increased sweating is characterized by local changes in salt balance. Human skin undergoes a significant change in pH when treated with detergents, and when the skin is exposed to sunlight, a significant amount of free radicals are formed.

#### 3.3.1. Osmotic Stress

Bacterial cells of control and experimental populations of both strains were incubated for 2 h in the presence of 33% NaCl solution. Gram-negative bacteria were found to be more resistant to osmotic stress. The cell number in the control population of Gram-positive bacteria *M. luteus* decreased by 21 times from (2.3 ± 0.1) × 10^8^ to (1.1 ± 0.1) × 10^7^, and for control population of *A. faecalis* the cell number decreased only by 1.8 times from (7.8 ± 0.3) × 10^8^ to (4.3 ± 0.1) × 10^8^ CFU/mL (Figure 4). The N and 10 N populations of both strains were more tolerant to high salt concentrations. The number of cells increased by 146 and 127% in *M. luteus* (Figure 4a) and by 128 and 151% in *A. faecalis* (Figure 4b) for N and 10 N, respectively, compared with the control. As with thermal stress, the N Gram-negative bacteria population and the 10 N Gram-positive bacterial population demonstrated the highest resistance. Bacterial osmotic resistance of the 100 N populations of both strains remained almost on the same level as it was for control. *M. luteus* grown in the presence of the highest concentrations of the peptide (populations 1000 N and 10,000 N) showed a higher tolerance to osmotic stress in terms of the CFU number. These values were 18 and 36% higher compared to the control. A slightly different picture has been observed for *A. faecalis*. The cell number decreased by 28% after 2 h of NaCl exposure for the 1000 N population, and for the 10,000 N population it remained almost at the same level as for the control population.

#### 3.3.2. Oxidative Stress

To study the oxidative effect, bacterial cells were exposed to 0.3% of hydrogen peroxide. It turns out that the Gram-negative strain was more sensitive to this type of stress. The number of cells in the control population of *A. faecalis* decreased by more than two orders of magnitude, while for the control population of *M. luteus*, the decrease in the cell number did not exceed ten times (Figure 4).

All experimental populations of *M. luteus* showed higher tolerance to oxidative stress than the control. The 10 N and 10,000 N populations had the highest resistance to this type of stress; the cell numbers were 137 and 187% of the control (Figure 4a).

Among all experimental populations of *A. faecalis*, 1000 N was the most resistant to oxidative stress. The number of cells that survived exposure to hydrogen peroxide increased by 60% compared to the control. For cells growing in the presence of N and 10 N peptide concentrations, the cell number decreased by 28 and 36% compared to the control (Figure 4b).

#### 3.3.3. Acid and Base Stresses

The study of the effect of BNP on the ability of bacteria to tolerate abrupt changes in the pH of the medium, both upward and downward, was also carried out using cells of the exponential growth phase. For this purpose, HCl was added to the control and experimental bacterial populations to lower the pH to 3.5 or NaOH to increase it to 10.5. After 24 h of incubation, the cell numbers were counted (Figure 4). Gram-positive strain *M. luteus* appeared to be more sensitive to the increase of the pH value up to 10.5, and Gram- negative *A. faecalis* was less resistant to its reduction down to 3.5.

Significant differences in resistance to pH changes were observed only for experimental populations growing in the presence of high concentrations of BNP (1000 N and 10,000 N). Under both acid and alkaline exposure, the number of cells in these populations was invariably about 1.5–1.6 times higher compared to the control (Figure 4a).

The N, 10 N, and 100 N populations of *A. faecalis* suffered a significant decrease (by 5.8, 7.5, and 17.3 times, respectively) in the cell numbers under acid stress, while at higher BNP concentrations, a greater resistance to pH reduction compared to the control was observed. When exposed to high pH stress, all experimental populations survived better than the control, but only by 7–20% (Figure 4b).

### 3.4. Effect of BNP on Antibiotic Resistance in Bacterial Cells

One of the most important adaptive abilities of human symbiont bacteria is their resistance to antibiotic treatment. Antibiotics taken by the patient during treatment have a strong effect on his microbiota, killing not only pathogenic bacteria, but also beneficial microorganisms that inhabit the intestines.

This study shows differences in antibiotic resistance between control and experimental (grown in the presence of BNP) populations of *M. luteus* and *A. faecalis*. The antibiotic resistance of bacteria was assessed by the values of the minimum inhibitory concentrations (MIC) of two antibiotics, ciprofloxacin and tetracycline (Table 2).

Almost all experimental populations tolerated the effect of ciprofloxacin and tetracycline better by about 1.2–1.7 times. The only exception was the N population, which appeared to be more sensitive to tetracycline treatment than the control. The results showed a trend: the effect of antibiotic resistance was proportional to the concentration of the peptide used. Ciprofloxacin’s MIC was 12.5 µg/mL for the control population of *M. luteus*, and MIC values for experimental populations were 22%, 36%, 42%, 48%, and 65% higher for N, 10 N, 100 N, 1000 N, and 10,000 N, respectively (Table 2). A similar trend and range of MIC values were noted in experiments with Gram-negative bacteria *A. faecalis*.

## 4. Discussion

Thus, our study showed that the presence of BNP in the growth medium of human symbiont bacteria affects their growth characteristics, survival, and stress resistance, including antibiotic resistance. In this case, the direction and magnitude of the effects depend on the type of bacteria, their growth conditions, and the concentration of the peptide.

The short half-life of the peptide of 20–40 minutes refers to the human body, where it is rapidly degraded [22,23]. We believe, based on the studies of other authors [18,19,21,35,43,44], that in our experiments the peptide molecules persist much longer, since there are no factors for their destruction. The action of the peptide, which affects the survival of cells during long-term storage, can be explained by the fact that it regulates the time of expression of the necessary genes in response to the peptide level. This happens during population growth. That is, cells grown in the presence of the peptide have a somewhat different set (or content) of proteins, which subsequently, during long-term storage, helps them survive, or vice versa. In this work, we only assessed the effect of the peptide on stress resistance and cell survival under various types of exposure, but did not analyze in detail the mechanisms that are of great interest and should be considered in further studies.

Comparing the results obtained for Gram-positive and Gram-negative bacteria, we can conclude that the effects of BNP on bacterial growth were different, while the effects on cell survival and stress resistance were largely similar. Thus, in the experiments, a noticeable dose-dependent effect of the peptide on the growth characteristics of Gram-positive bacteria *M. luteus* was revealed. It has been shown that the cultivation of micrococcus in the presence of BNP at low doses (N and 10 N, or 25 and 250 pg/mL), as well as at very high doses (10,000 N or 250,000 pg/mL) stimulates cell growth and promotes their survival during long-term storage. At the same time, the medium concentration of the peptide (100 N, or 2500 pg/mL), corresponding to the hormone level in a person suffering from an extreme degree of pathology of the cardiovascular system, had a lesser effect on the growth characteristics of bacteria and more than two times reduced the viable cell number after a month of storage.

On the other hand, BNP had no significant effect on the growth of Gram-negative *A. faecalis* under microaerophilic conditions. These data are consistent with the results of other researchers. For example, Lesouhaitier et al. showed that BNP does not modify the growth or culturability of *P. fluorescens* MF37 [43], but can modulate the synthesis of some *Pseudomonas aeruginosa* molecules sensitive to quorum sensing [44]. However, our experiments showed that the effect of the peptide on the growth characteristics of both Gram-positive and Gram-negative bacteria also largely depends on the choice of cultivation conditions, the variation of which can change the effect induced by the peptide. At the same time, the less optimal the conditions for bacterial growth, the more noticeable the effect of BNP. This once again emphasizes the extreme flexibility of the mechanisms of adaptation of bacteria to environmental conditions.

The demonstrated ability of BNPs to increase (by 1.3–1.7 times) the number of surviving cells during long-term (up to 4 months) storage of both Gram-positive and Gram-negative bacteria can be used in practice. For example, industrial and municipal wastewater treatment plants can suffer from inefficiency or failure caused by overloading or toxic shock. Use of microbial preparations cultured in the presence of BNP could ensure enhanced microbial activity in such wastewater treatment plants. Biological products are being widely used in organic agriculture to increase yields [45] or protect plants from diseases caused by various pathogens [46]. For example, *A. faecalis* is considered a plant growth-promoting rhizobacteria (PGPB) because of its ability to produce indole acetic acid (IAA) and 1-aminocyclopropane-1-carboxylic acid (ACC) deaminase, solubilize phosphates, and fix atmospheric nitrogen [47,48]. However, drought, salinity and other important environmental factors can not only hamper agricultural productivity, but also decrease the effectiveness of such biological preparations. A relatively new area is also the use of probiotics in cosmetology and personal care products [49]. However, increased sweating, characterized by local changes in salt balance, a significant change in pH when treated with detergents, and a significant amount of free radicals when exposed to sunlight can reduce the effectiveness of such probiotics on the skin. Thus, in addition to clinical medical practice, our discoveries can be used in the creation of biological products with increased resistance to environmental stress factors for various industries.

Also important for the production of bacterial preparations is the ability of the peptide to increase the thermal stability of cells, even those at the stage of exponential growth. It can be assumed that BNP regulates the expression of genes that protect against heat shock, and this regulation is highly dependent on the concentration of the peptide. It should be noted that the range of changes in the number of surviving cells in experimental populations compared to the control of all the stresses tested in this work was the most significant for heat shock. The number of surviving cells in the experimental populations could differ from the control up to 15 times or even be equal to 0 (100 N and 1000 N in *A. faecalis*). For both *M. luteus* and *A. faecalis*, increased cell heat tolerance was observed at low concentrations of BNP (healthy human peptide level) and medium concentrations (sick human peptide level) showed the lowest heat tolerance. A high dose of the peptide again contributed to an increase in the heat tolerance of the bacteria.

Usually, most studies related to the effect of HRF on human symbiont bacteria are aimed at studying changes in their virulence, antibiotic resistance, and the ability to form persister cells or biofilms [50,51,52,53]. For example, in Ref. [49], it was noted that BNP and CNP strongly decrease *P. aeruginosa* biofilm formation. In our work, it was also shown that in almost all experimental populations, BNP increases the resistance of both Gram-positive and Gram-negative bacteria to the action of two antibiotics, tetracycline and ciprofloxacin. This followed with an increase in direct proportion to the peptide concentration of the MIC values for experimental populations compared to the control. Therefore, it can be assumed that in a human body with cardiovascular disease, bacterial cells are more resistant to antibiotics.

In addition to temperature and antibiotic stress, we have for the first time studied the effect of BNP on several other major types of stress that bacteria in the human body can face, such as osmotic, oxidative, acidic, and alkaline. The effect of the peptide to a greater or lesser extent affected the resistance of bacteria to all of these four stresses. For Gram-positive *M. luteus* cells, the effect of BNP was noticeable during osmotic and oxidative shock. Moreover, in most cases, an increase in cell resistance to all four stress factors was observed in experimental populations cultivated in the presence of both the lowest concentrations of BNP (N or 10 N) and the highest (10,000 N), and a decrease in cell resistance was observed in the 100 N population. For Gram-negative *A. faecalis* cells, the effect of BNP was especially noticeable under oxidative and acid stress. In these cases, in contrast to osmotic and alkaline stress, the cells of the N and 10 N populations, on the contrary, were more sensitive than the control. At the same time, in the 10,000 N populations, the resistance of bacteria was higher than the control.

Our findings once again confirm the fact that the action of BNP is dose-dependent and is realized through different regulatory mechanisms. Further work will be devoted to studying the mechanisms of the observed effects.

## 5. Conclusions

Our findings once again confirmed the dependence of the activity of the bacterial microflora that develops on the skin or in the human intestine on the level of humoral regulation factors (HRF). Bacterial populations that develop in the healthy human body at a BNP content of up to 250 pg/mL will be very different from the microbiota of a person with heart failure and a person with an extreme degree of pathology of the cardiovascular system, when the peptide content is up to 2500 pg/mL. The main conclusion of the work is that bacterial populations that develop in the healthy human body will be more stress-resistant than in a person suffering from extreme heart failure.

The mechanism of changes in the adaptive responses of bacteria seems to be associated with the regulation of the expression of the necessary genes in response to the host HRF level. Using this mechanism, bacteria can adapt to any stressful situation: increase resistance to high concentrations of salts, oxidizing agents or antibiotics, survive exposure to thermal or pH shock.

A wide range of BNP concentrations studied in this work made it possible to evaluate the potential of this peptide action on bacteria. The results obtained are promising to be used both for scientific purposes to expand the understanding of the adaptive capabilities of bacteria, and to solve practical problems of obtaining cells with increased resistance to environmental stress. The patterns shown for the effect of BNP on stress resistance, including antibiotic resistance, of Gram-negative bacteria are particularly important for clinical use and may help in the treatment of people with cardiovascular diseases. Additionally, the shown ability of BNP to stimulate growth and increase the stress resistance of bacterial cells can be used in the production of bacterial preparations for agriculture, cosmetology, and waste management.

## Figures and Tables

**Table 1:** Influence of BNP on the activity of *M. luteus* and *A. faecalis* under aerobic and microaerophilic conditions.

Population	*M. luteus*, after Daily Incubation			*A. faecalis*, after Daily Incubation		
	CO_2_ under Aerobic Conditions, in %	Under Microaerophilic Conditions		CO_2_ under Aerobic Conditions, in %	Under Microaerophilic Conditions	
		CO_2_, in %	Acetate, in mg/L		CO_2_, in %	Acetate, in mg/L
Control	9.55 ± 0.25	12.81 ± 0.21	2.29 ± 0.08	13.7 ± 0.18	12.5 ± 0.15	0.21 ± 0.02
N	10.25 ± 0.44	12.96 ± 0.15	2.52 ± 0.09	13.7 ± 0.22	13.0 ± 0.12	1.36 ± 0.07
10 N	10.78 ± 0.41	13.7 ± 0.19	3.61 ± 0.10	13.8 ± 0.19	13.6 ± 0.26	1.65 ± 0.11
100 N	9.87 ± 0.12	13.4 ± 0.26	3.21 ± 0.11	13.7 ± 0.17	13.3 ± 0.21	1.53 ± 0.10
1000 N	11.31 ± 0.37	12.93 ± 0.23	3.21 ± 0.05	13.8 ± 0.20	13.1 ± 0.09	0.74 ± 0.08
10,000 N	11.74 ± 0.20	13.18 ± 0.13	3.19 ± 0.06	13.8 ± 0.27	12.8 ± 0.14	0.42 ± 0.04

**Table 2:** MIC values of ciprofloxacin and tetracycline for *M. luteus* and *A. faecalis* bacterial cells of control and experimental populations.

Population	MIC, µg/ mL, (in % of Control)			
	Ciprofloxacin		Tetracycline	
	*M. luteus*	*A. faecalis*	*M. luteus*	*A. faecalis*
Control	12.5 ± 0.4 (100)	16.3 ± 0.2 (100)	7.3 ± 0.3 (100)	30.0 ± 0.3 (100)
N	15.3 ± 0.3 (122.4)	20.7 ± 0.4 (127.0)	6.0 ± 0.4 (82.2)	24.8 ± 0.4 (82.7)
10 N	17.1 ± 0.4 (136.8)	22.0 ± 0.4 (135.0)	8.5 ± 0.2 (116.4)	32.0 ± 0.2 (106.7)
100 N	17.8 ± 0.2 (142.4)	22.4 ± 0.3 (137.4)	9.1 ± 0.4 (124.7)	35.9 ± 0.4 (119.7)
1000 N	18.5 ± 0.5 (148.0)	24.5 ± 0.1 (150.3)	10.6 ± 0.4 (145.2)	36.5 ± 0.5 (121.7)
10,000 N	20.6 ± 0.3 (164.8)	25.9 ± 0.6 (158.9)	12.5 ± 0.3 (171.2)	40.2 ± 0.6 (134.0)

## Data Availability

Not applicable.
